# The use of transformed IMR90 cell model to identify the potential extra-telomeric effects of *hTERT* in cell migration and DNA damage response

**DOI:** 10.1186/1471-2091-15-17

**Published:** 2014-08-07

**Authors:** Xu Cao, Chiou Mee Kong, Kanchi Madhu Mathi, Yoon Pin Lim, Valere Cacheux-Rataboul, Xueying Wang

**Affiliations:** 1 Department of Biochemistry, Yong Loo Lin School of Medicine, National University of Singapore, Block MD4, Level 1, 5 Science Drive 2, Singapore 117545, Singapore; 2 Gene Regulation Laboratory, Genome Institute of Singapore, Singapore, Singapore; 3 National University Cancer Institute of Singapore (NCIS), Singapore, Singapore

**Keywords:** Extra-telomeric, hTERT, Neoplastic transformation, Cell migration, DNA damage response

## Abstract

**Background:**

Human telomerase reverse transcriptase (hTERT), the catalytic subunit of telomesase, is responsible for telomere maintenance and its reactivation is implicated in almost 90% human cancers. Recent evidences show that hTERT is essential for neoplastic transformation independent of its canonical function. However, the roles of hTERT in the process remain elusive. In the current work, we explore the extra-telomeric role of hTERT in the neoplastic transformation of fibroblast IMR90.

**Results:**

Here we established transformed IMR90 cells by co-expression of three oncogenic factors, namely, H-Ras, SV40 Large-T antigen and hTERT (RSH). The RSH-transformed cells acquired hallmarks of cancer, such as they can grow under anchorage independent conditions; self-sufficient in growth signals; attenuated response to apoptosis; and possessed recurrent chromosomal abnormalities. Furthermore, the RSH-transformed cells showed enhanced migration capability which was also observed in IMR90 cells expressing hTERT alone, indicating that hTERT plays a role in cell migration, and thus possibly contribute to their metastatic potential during tumor transformation. This notion was further supported by our microarray analysis. In addition, we found that Ku70 were exclusively upregulated in both RSH-transformed IMR90 cells and hTERT-overexpressing IMR90 cells, suggesting the potential role of hTERT in DNA damage response (DDR).

**Conclusions:**

Collectively, our study revealed the extra-telomeric effects of hTERT in cell migration and DDR during neoplastic transformation.

## Background

Human telomeres are TTAGGG repeats at the ends of human chromosomes, protecting them from end-to-end fusion and maintaining chromosomal stability [1,2]. In human somatic cells, telomere length shortens in each cell division as DNA polymerase is unable to replicate the very end of telomere [3,4]. Eventually cells stop proliferating and undergo senescence. However, almost 90% of human cancers overcome the finite divisional potential by reactivation of a ribonucleoprotein complex, known as telomerase [5-7]. The catalytic subunit of telomerase, telomerase reverse transcriptase (hTERT), is the rate-limiting factor of telomerase activity. Up-regulation of hTERT confers cells limitless proliferative potential, which is one of the cancer hallmarks [8].

Emerging evidence suggests that maintenance of telomere length might not the sole function of hTERT during oncogenesis. For instance, knockdown of hTERT resulted in rapid inhibition of cell proliferation and growth in cancer cells without affecting the telomere length [9]. Moreover, overexpressing hTERT in mice with long telomeres attributed to increase risk of cancer formation [10], which further support the notion that hTERT has other functions, on top of its canonical function in telomere maintenance. In addition, human cells utilize alternative lengthening of telomeres pathway for telomere maintenance can only be transformed when co-expressing hTERT and oncogenic-Ras, indicating that hTERT is indispensable for cancer transformation [11,12]. Taken together, these findings suggest that hTERT plays a key role in tumorigenesis independent of its canonical function. Its roles in neoplastic transformation, however, are still not well understood. Therefore, investigating the roles of hTERT in the cancer transformation is of utmost imperative.

Neoplastic transformation can be achieved by *in vitro* genetic manipulation. Studies showed that disruption of the intracellular pathways regulated by SV40 Large-T, oncogenic Ras and hTERT are sufficient to create a human tumor cell [13]. This highlighted the various pathways that require changes for transformation to occur: the mitogenic response pathway activated by Ras [14]; telomere maintenance pathway by hTERT [4]; cell surveillance pathways due to the functional abolishment of p53 and Rb tumor-suppressors by Large-T [15]. Since disruption of these cellular pathways are commonly seen in *in vivo* tumors, tumor cells generated from such transformed cell model can be a good representation of actual human cancers [16]. This model also serves as a platform to study the early stages of the tumor formation, as compared to tumor biopsies that are often obtained at an advanced stage [13].

Here, we transformed IMR90, a non-epithelial somatic lung fibroblast, by three factors, including H-Ras, SV40 Large-T, and hTERT (RSH). Using the RSH-transformed IMR90 cell model, our results unveiled the extra-telomeric functions of hTERT in cell migration as well as in DNA damage response during neoplastic transformation. Therefore, our findings suggest that hTERT is an attractive target for cancer therapy, even at early stage of cancer formation.

## Results and discussion

### RSH-transformed cells acquire cancer cells characteristics

**Figure 1:**
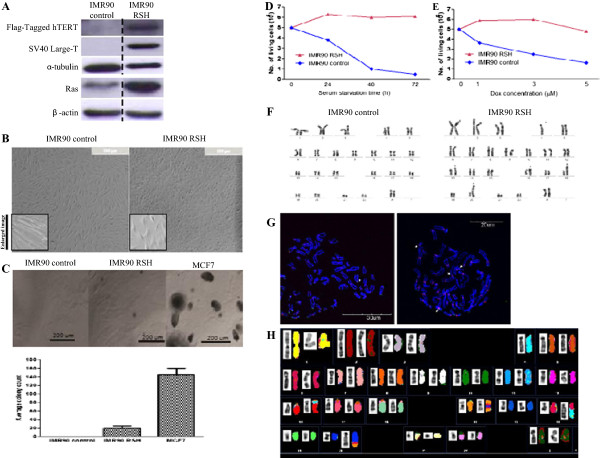
**Transformed IMR90 cells show characteristics of a cancer cell. (A)** Western blot confirming the expression of the three genetic factors Ras, hTERT and SV 40 Large T in the transformed IMR90 primary human cells. The expression of hTERT on the western blot was detected using anti-FLAG antibody. **(B)** Changes in cellular morphology after RSH transformation. Transformation of IMR90 cells and resulted in shorter and rounder cells. Left bottom corners show the enlarged pictures. **(C)** Soft agar assay determining the anchorage independence of the transformed RSH cells *in vitro*. MCF-7 cells were used as the positive control. The experiment was carried out in triplicates. Representative image of one well is shown. **(D)** Survival of IMR90 control and IMR90 RSH cells in serum-free medium. **(E)** Cells of IMR90 control and IMR90 RSH were treated with 1 μM, 3 μM and 5 μM of doxorubicin separately for up to 48 hours. **(F)** G-band Karyotype analysis of IMR90 control cells infected with control vector and IMR90 RSH cells. Arrows indicate the presence of genetic aberrations. **(G)** Metaphase spreads of IMR90 RSH cells. White arrows indicate the presence of genetic aberrations. **(H)** A representative spectral karyotype of a metaphase from IMR90 RSH cells. Recurrent abnormality is defined as at least 3 metaphase cells having the abnormality at the same region of chromosomal location. Chromosomal abnormality can be observed at chromosomes 4, 18, 20.

Primary human fibroblast cells IMR90 were successfully co-transfected with Ras, SV40 Large-T, and hTERT and their protein expressions were confirmed by western blotting (Figure 1A). Morphologically, IMR90 RSH fibroblasts appeared to be shorter and rounder compared to the infection control (Figure 1B). This observation is consistent with the findings of Mason and colleagues in IMR90 cells transformed with E1a/Ras [17], suggesting that these changes are the unique characteristics of cellular transformation. Moreover, late passages of IMR90 control cells underwent significant increase in cell sizes, indicating their senescent status. However, this was not observed in IMR90 RSH cells even after several passages (data not shown).

One of the hallmarks of the cancerous cells is they can survive and grow in the absence of anchorage to the extracellular matrix [18]. Our anchorage independent growth assays demonstrated that IMR90 RSH cells formed small microscopic colonies (<200 μm in diameter) while MCF-7 cells, the positive control, formed large visible colonies (>200 μm in diameter) (Figure 1C) after 6 weeks. Comparison of colony sizes with MCF-7 suggests that transformation by three genetic factors produced cells that were less tumorigenic than the established cancer cells. Thus, the RSH-transformed cell could serve as a representative model to study the early events of cancer transformation, compared to an established cell line.

Another common trait of cancer cells is their self-sufficiency in growth signals [18]. To investigate the effects of growth factors withdrawal on the RSH-transformed cells, the cells were subjected to a serum-free environment, following which cell proliferation and survival were assessed over a three-day period. As expected, IMR90 control cells showed signs of apoptosis after 24 hours of serum withdrawal. However, no apoptosis was observed in IMR90 RSH cells even after treating for 72 hours in serum-free condition, suggesting that these RSH-transformed cells were able to survive in the absence of growth factors (Figure 1D). We further tested whether transformed fibroblasts are refractory to the induction of apoptosis, which is commonly observed in cancer cells [19]. After treating with Doxorubicin (Dox), a DNA damage-inducing drug for 48 hours, IMR90 RSH cells were able to survive even at much higher Dox concentrations (3 μM and 5 μM) than IMR90 control cells, reflecting an attenuation in the apoptotic machinery of the cells (Figure 1E).

For potential use of IMR90 RSH as a cancer cell model, we also assessed their chromosomal aberrations for recurrent abnormalities through cytogenetics study [20]. Karyotyping of IMR90 RSH cells revealed recurrent abnormalities (Figure 1F,G). Moreover, spectral karyotyping analysis revealed that 60.9% (14 out of 23) of IMR90 RSH cells had recurrent chromosomal abnormalities at chromosomes 4, 18, 20 (Figure 1H; Additional file 1: Table S1). Taken together, these data indicated that IMR90 cells were successfully transformed by co-expressing three oncogenic factors. The RSH-transformed fibroblasts acquired various characteristics of a human cancer cell and may serve as a valuable model to study the early events of tumorigenesis.

### RSH-transformed cells and hTERT-overexpressing cells demonstrate increased migration capability

**Figure 2:**
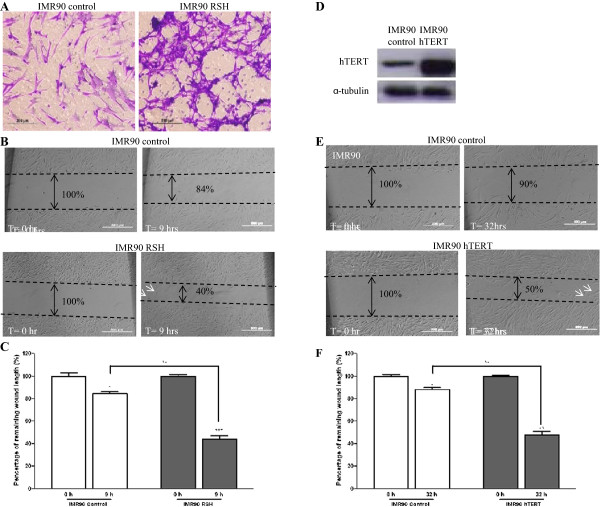
**Migration capability analysis of IMR90 RSH and IMR90 hTERT cells. (A)** Boyden assay comparing the migration capability of IMR90 control and IMR90 RSH cells after 10 hours. **(B)** Wound healing assay comparing the migration of IMR90 control and IMR90 RSH cells after 9 hours of incubation. Images at 0 hour and at 9 hours, representative of triplicate experiments for IMR90 control and IMR90 RSH cells, are shown. White arrows indicate individual cells that have migrated. **(C)** The ‘wound closure’ areas are visualized under an inverted microscope and bar graphs show the distance travelled by IMR90 control and IMR90 RSH cells in the wound healing assay. Results are indicated as the mean ± standard deviation (SD) (n = 2). *p < 0.05; **p < 0.01; ***p <0.001. **(D)** Western blot confirming the overexpression of hTERT in IMR90 primary human cells. **(E)** Wound healing assay comparing the migration of IMR90 control and IMR90 hTERT cells after 32 hours of incubation. Images at 0 hour and at 32 hours, representative of triplicate experiments for IMR90 control and IMR90 hTERT cells, are shown. White arrows indicate individual cells that have migrated*.***(F)** Bar graphs showing the distance travelled by IMR90 control and IMR90 hTERT cells in the wound healing assay. Results are indicated as the mean ± standard deviation (SD) (n = 2). *p <0.05; **p < 0.01; ***p <0.001.

Metastasis is often correlated to two attributes: migration followed by invasion [21]. Metastatic tumors are believed to occur in the late stages of cancer [22]. However, in numerous early-stage cancers, recurrence can be observed even after the removal of non-invasive, benign tumors, suggesting the possibility of the cancer cells undergoing metastasis at a much earlier stage [23]. We questioned if IMR90 RSH cells possess migration capability similar to that of a cancer cell. Our Boyden assay results showed that IMR90 RSH cells had a greater migration capability as compared to IMR90 control cells (Figure 2A). This result was further validated with wound healing assay, in which the gap was reduced to 40% for IMR90 RSH cells compared to 84% in IMR90 control cells (*p* < 0.01) (Figure 2B,C). Moreover, we observed that IMR90 RSH cells migrated faster and in a more individualistic pattern compared to IMR90 control cells, implying some degree of autonomy (Figure 2B).

Given that transformation can increase the migration capability of cells and that hTERT is one of the upregulated factors in the transformed cells, it then raised the question as to whether hTERT alone can contribute to this phenomenon. In order to assess the possible role of telomerase in cell migration, we also performed wound healing assay on IMR90 cells expressing hTERT alone. Similar to IMR90 RSH cells, the hTERT-overexpressing IMR90 cells (Figure 2D) also migrated faster than IMR90 control cells (Figure 2E). However, when compared to IMR90 RSH cells, the migration process in IMR90 hTERT cells started to occur only after 10 hours and they seemed to have a slower migration rate. As shown in Figure 2E and F, IMR90 hTERT cells migrated about 50% distance, yet the distance was only reduced by 10% for IMR90 control cells (*p* < 0.01). Collectively, these suggest that hTERT does play a role in cell migration, which in turn contributes to the metastatic potential of cancer cells.

### Microarray analysis supports the notion that hTERT plays a role in cell migration

To investigate the underlying molecular basis of hTERT-induced migration in transformed cells, we analyzed the genome-wide gene expression in IMR90 RSH cells and IMR90 hTERT cells. There were a total of 62 and 150 migration-related genes that were found to be differentially expressed (fold change ≥ 2; *p* < 0.05) in IMR90 RSH cells and IMR90 hTERT cells respectively. Among these differentially expressed genes, 28 genes were found to be overlapped in both IMR90 RSH and IMR90 hTERT cells (Table 1), suggesting that these genes may attribute to migration during neoplastic transformation and specifically, they were under the regulation of hTERT. Approximately 93% of the genes were found to be up-regulated by hTERT. Remarkably, pro-inflammatory factors such as Interleukin-6 (IL6) and IL8 were significantly enhanced in both RSH-transformed cells and hTERT-overexpressing cells. Previous published data demonstrated that IL6 and IL8 are targets under hTERT-mediated regulation of NF-κB pathway [24]. On top of their role in inflammation, they are also found to be involved in tumor cell migration and invasion [25], which is corroborated with our microarray data. Taken together, it is reasonable to hypothesize that hTERT could enhance the cell migration through NF-κB pathway in early tumorigenesis.

**Table 1:** Changes in migration-related genes in both IMR90 RSH and IMR90 hTERT cells

**Gene symbol**	**Gene name**	**Function on cell migration**	**Fold change**	
			**IMR90 RSH vs control**	**IMR hTERT vs control**
IL6	Interleukin 6	Positive regulation of cell migration	14.135	2.665
APOE	Apolipoprotein E	Negative regulation of cell motion	7.82747	5.968
COX-2	Cyclooxygenase-2	Positive regulation of cell migration	6.094	2.475
LAMA5	Laminin, alpha 5	Positive regulation of cell migration	4.273	2.498
IL8	Interleukin 8	Positive regulation of locomotion	3.755	2.588
BTG1	B-cell translocation gene 1	Positive regulation of cell migration	3.34	3.392
SCG2	Secretogranin II	Positive regulation of locomotion	3.051	3.563
ETV4	Ets variant 4	Positive regulation of cell migration	3.047	2.884
CKLF	Chemokine-like factor	Positive regulation of cell migration	2.865	3.246
COL18A1	Collagen, type XVIII, alpha 1	Positive regulation of cell migration	2.738	4.533
LAMA1	Laminin, alpha 1	Positive regulation of cell migration	2.631	6.232
LAMA4	Laminin, alpha 4	Positive regulation of cell migration	2.582	2.3
MYH10	Myosin, heavy chain 10, non-muscle	Positive regulation of cell migration	2.495	5.678
BBS2	Bardet-Biedl syndrome 2	Positive regulation of cell migration	2.479	2.415
WASF2	WAS protein family, member 2	Positive regulation of cell migration	2.421	5.506
TWIST1	Twist homolog 1 (Drosophila)	Positive regulation of cell migration	2.409	3.328
NR4A2	Nuclear receptor subfamily 4, A2	Positive regulation of cell migration	2.398	3.488
SMAD3	SMAD family member 3	Positive regulation of cell migration	2.396	3.602
PLAT	Plasminogen activator, tissue	Positive regulation of cell migration	2.367	3.977
SEMA3F	Semaphorin 3 F	Positive regulation of cell migration	2.348	5.71
NUP85	Nucleoporin 85 kDa	Positive regulation of cell migration	2.214	3.579
PALM	Paralemmin	Positive regulation of cell migration	2.151	2.42
ROBO3	Roundabout, axon guidance receptor, homolog 3	Positive regulation of cell migration	2.116	2.929
NDE1	nudE nuclear distribution gene E homolog 1	Positive regulation of cell migration	2.115	2.388
CDKN1B	Cyclin-dependent kinase inhibitor 1B	Negative regulation of cell motion	2.068	2.316
LYN	v-yes-1 Yamaguchi sarcoma oncogene	Positive regulation of cell motion	2.032	4.017
PRKDC	Protein kinase, DNA-activated, catalytic polypeptide	Positive regulation of cell migration	2.024	3.337
ACTG1	Actin, gamma 1	Positive regulation of cell migration	-5.041	-3.107

On the other hand, Liu *et al.* in 2013 have shown that hTERT promotes the epithelial-mesenchymal transition (EMT) by upregulating snail family zinc finger 1 (Snail1) and vimentin through Wnt/beta-catenin signaling pathway in gastric cancer [26]. We did not observe significant changes in Snail-1 and vimentin genes expressions in our cell models and this could be attributed to different cell model used in both studies. However, we noticed that two downstream genes of Wnt/beta-catenin pathways, namely Cyclooxygenase-2 (COX-2) and Twist1 were significantly enhanced in both RSH-transformed cells and hTERT-overexpressing cells. Both were reported to be implicated in cancer cell migration [27,28], and this is in consistent with our microarray data. Taken together, our microarray data complements with our previous notion that hTERT play an important role in cell mobility, which in turn contributes to the metastatic potential of cancer cells.

### hTERT might be implicated in DNA damage response via upregulating Ku70 during IMR90 transformation

**Figure 3:**
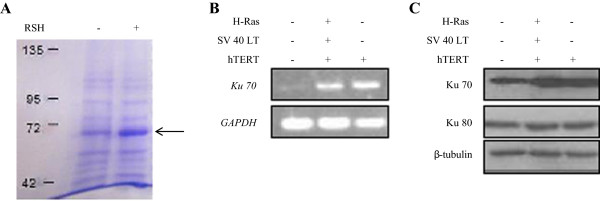
**Ku 70 expression in IMR90-RSH and IMR90 hTERT cells. (A)** Coomassie blue staining of the protein expression in RSH transformed cells and control fibroblasts, showing an augmented ~70 kDa (indicated by the black arrow). **(B)** Semi-quantitative analysis of Ku70 by RT-PCR in IMR90 control, IMR90 hTERT and IMR90 RSH cells. Ku70 expression showed an increase in IMR90 RSH and IMR90 hTERT cells compared to IMR90 control cells. **(C)** Ku70 protein expression in IMR90 control, IMR90 hTERT and IMR90 RSH cells. Ku70 protein expression showed an increase in IMR90 RSH and IMR90 hTERT cells compared to IMR90 control cells.

To delineate the roles of hTERT in neoplastic transformation, we performed mass spectrometry analysis on the protein levels of IMR90 RSH cells. Surprisingly, analysis by mass spectrometry revealed that protein Ku70 was exclusively found in IMR90 RSH cells, but not in IMR90 control cells (Figure 3A; Additional file 2: Table S2). Moreover, both RT-PCR and immunoblotting results confirmed our observation where Ku70 expression was augmented in IMR90 RSH as well as IMR90 hTERT cells, but not in IMR90 control cells (Figure 3B,C). This could be indicative of an activated DNA damage responses (DDR) in both IMR90-RSH and IMR90-hTERT since Ku70 is a DDR sensor and is implicated in the non homologous end joining (NHEJ) pathway [29]. Ku70 forms a heterodimeric complex with Ku80 [30]. However, the protein expression of Ku80 remained unchanged (Figure 3C). Presence of Ku70 in IMR90 RSH cells and IMR90 hTERT cells prompted us to assess whether other DDR-associated proteins could be regulated by hTERT as well. Our microarray data revealed that several DDR-associated genes were upregulated in both RSH-transformed IMR90 and hTERT-overexpressing IMR90 cells (fold change ≥ 2; *p* < 0.05) (Additional file 3: Table S3). Taken together, these results suggest that hTERT may play an important role in DDR pathways, via Ku70 and other DDR-associated proteins.

## Conclusions

In conclusion, the neoplastic transformation of human primary fibroblast cells using SV40 Large-T antigen, H-Ras and hTERT can be used as an *in vitro* cell model for cancer studies, especially for those fibroblasts originated tumors. Moreover, our findings suggest that hTERT is implicated in cell migration as well as DDR during neoplastic transformation. As these processes have great implications in cancer progression, our study could provide insights on the roles of hTERT and its underlying mechanisms in human cancer formation.

## Methods

### Cell lines

Human breast cancer cells MCF-7 (HTB-22) and normal human fibroblasts, including IMR90 (CCL-186) and BJ (CCL-186) were purchased from American Type Culture Collection (ATCC). Cells were cultured in Dulbecco’s Modified Eagle’s Medium (DMEM) supplemented with 10% fetal bovine serum (FBS) (Gibco, Invitrogen), 2 mM L-glutamine (Gibco, Invitrogen) and 100U/ml penicillin and streptomycin (Gibco, Invitrogen).

### Infection and selection of cells

Retroviral pBabe-puro Ras V12 (Addgene Plasmid 1768), and retroviral pBabe-puro SV40 LT (Addgene Plasmid 13970) were purchased from Addgene. FLAG was tagged to the N-terminus of hTERT and cloned into a lentiviral vector with Ires hygromycin mammalian selection. Retroviral pBabe-puro-hTERT vector (Addgene Plasmid 1771) was used to generate hTERT-overexpressing cell. Retroviral pBabe-puro vector (Addgene Plasmid 1764) was used as infection control. IMR90 was infected with virus supernatant with 8 μg/ml of polybrene for 24 hours. To generate stable cell line that co-expresses Ras, SV40 Large T and hTERT, cells infected with RSH combination were selected using both puromycin and hygromycin (Invitrogen) for 5 days. Cells infected with pBabe-puro-hTERT as well as pBabe-puro control vector were selected using puromycin (InvivoGen) for 5 days. Cells stably expressing the desired genes were further passaged and maintained on selection medium for an additional two to three weeks prior to downstream experiments.

### Anchorage-independent growth assay

10^4^ cells were seeded in 0.3% (w/v) agarose with DMEM and 16.66% FBS onto each well of a 24-well plate with a 0.6% (w/v) agarose underlay. Wells were analyzed for colony formation after 6 weeks. Scoring was done by counting the colonies under the microscope.

### Serum-free cell survival assay

10^5^ cells were seeded and the number of living cells was determined by Countess automated cell counter (Invitrogen) after treating them in serum-free DMEM for 24, 48 and 72 hours.

### Drug treatment

10^5^ cells were treated with Doxorubicin (Calbiochem) at 1 μM, 3 μM and 5 μM concentrations. The number of living cells was determined by cell counting 24 and 48 hours after drug treatment.

### Immunoblotting

Immunoblotting was performed as described previously [31]. The following antibodies were used: H-Ras (Santa Cruz), SV40 Large-T (Santa Cruz), FLAG-Tag (Sigma), hTERT (Epitomics), Ku 70 (Cell Signaling), Ku 80 (Cell Signaling), α-tubulin (Sigma) and β-actin (Abcam).

### Wound healing assay

6-well plates were first coated with 10 μg/ml of collagen (Cohesion) for two hours, followed by blocking with BSA for one hour. 5 × 10^5^ cells were seeded into the wells and allowed to grow until confluent. Following which, the cell monolayer was scratched at the bottom of the wells. Culture medium was then replaced with serum-free DMEM to minimize cell proliferation. Wells were observed under the microscope at different time-points and the distance of gap was measured. The percentage of wound closure was calculated from distances quantified in three independent experiments.

### Boyden assay

Migration assay was performed using cell culture inserts (BD Biosciences) with 8 μm pore size. 10^5^ cells were diluted in 300 μl of serum-free DMEM supplemented with 0.1% (w/v) BSA and added to the upper chamber of the well. 500 μl of normal culture DMEM supplemented with 100 ng/ml of human epidermal growth factor (ProSpec) was added to the lower chamber and incubated for 10 hours. Following which, medium was removed from both upper and lower chambers and cells were removed by swabbing from the upper chamber. Cells from the lower side of the insert were then stained with 1% crystal violet and observed under the microscope.

### Illumina microarray

Total RNA was extracted using RNeasy Mini Kit (Qiagen). 500 ng total RNA was used for cRNA amplification using Illumina TotalPrep RNA Amplification kit (Ambion), following manufacturers’ instructions. 750 ng cRNA was then hybridized onto the HumanRef-8 v2 Sentrix BeadChip (Illumina). Subsequently, the fluorescence emission by Cy3 was quantitatively detected by the Illumina BeadArray Reader software for downstream analysis of data by Partek software. Statistical significance of individual gene expression levels was analyzed by Analysis of variance (ANOVA) and significant genes were identified based on fold-changes. Threshold for significance was set at fold-change > ± |2.0| when compared to control cells. Absolute intensity differences between experimental groups were set at *p* < 0.05. All data is MIAME compliant and the raw data has been deposited in a MIAME compliant database (accession number: GSE24097).

### RT-PCR and DNA gel electrophoresis

cDNA was used in a total of 25 μL PCR reactions containing PCR buffer, DNA Taq polymerase, 200 nm of each primer and dNTP. PCR products were resolved using gel electrophoresis.

### Metaphase spreads

Metaphase spreads were prepared as described in Jeppesen’s protocol [32], with slight modifications. Slides were observed using the Olympus Fluoview 1000 confocal microscopy system.

### G-band karyotyping and spectral karyotyping

Cells at about 80% confluence were treated with colcemide for mitotic arrest and harvested by standard hypotonic treatment and methanol: acetic acid (3:1) fixation. For G-band karyotyping, slides were prepared by standard air drying method and G-band karyotype was performed according to the published protocols. For spectral karyotyping, slides were prepared by standard air drying method and hybridized with Human SKY paint probe (ASI), as per manufacturers’ recommendations. 23 metaphases were analyzed by SKY analysis. Identified chromosomal abnormalities were described according to the International System for Human Cytogenetic Nomenclature (ISCN) (1995). Recurrent abnormalities are defined as at least 3 metaphase cells having the abnormality at the same region of chromosomal location or those that are involved in > 3% of cases.

### Coomassie blue staining and mass spectrometry

Coomassie blue staining was performed on 7% and 15% SDS-PAGE gel. Bands were excised at 70 kDa and the spots were rehydrated in digestion buffer containing sequencing grade modified trypsin at 37°C. Digested peptides were extracted from gel with TFA extraction buffer and desalted using C-18 Zip-tips (Millipore). Mass spectra of the peptides in each sample were obtained by MALDI-TOF. Protein identification was based on peptide fingerprint mass mapping and peptide fragmentation mapping.

### Availability of supporting data

The data sets supporting the results of this article are available in the LabArchves. The unique persistent identifier and hyperlink to data sets are listed below:

http://www.ncbi.nlm.nih.gov/geo/query/acc.cgi?acc=GSE24097 (Microarray data)

https://mynotebook.labarchives.com/share/Xu%2520Cao/MjAuOHw0MDkwNC8xNi9UcmVlTm9kZS84MDAxMjY3Mjd8NTIuOA== (Additional file 1: Table S1)

https://mynotebook.labarchives.com/share/Xu%2520Cao/MjIuMXw0MDkwNC8xNy9UcmVlTm9kZS8zMjY0NzI2ODU1fDU2LjE= (Additional file 2: Table S2)

https://mynotebook.labarchives.com/share/Xu%2520Cao/MjMuNHw0MDkwNC8xOC9UcmVlTm9kZS80MTE5NjA3NzE4fDU5LjQ= (Additional file 3: Table S3)

## Abbreviations

hTERT: Human telomerase reverse transcriptase; RSH: H-Ras, SV40 Large-T antigen and hTERT; DDR: DNA, Damage response; Dox: Doxorubicin; IL6: Interleukin-6; IL8: Interleukin-8; COX-2: Cyclooxygenase-2; NHEJ: Non homologous end joining.

## Competing interests

All authors declare that they have no competing interests.

## Author’s contributions

XC, CMK, KMM and XW designed assays, performed experiments, and analyzed data. YPL and VCR performed experiments and aided in data analysis. XC, CMK, and XW contributed to the writing and editing of the manuscript. All authors read and approved the final manuscript.

## Supplementary Material

Additional file 1: Table S1 Summary of recurrent molecular cytogenetic abnormalities detected in IMR90 RSH cells. Click here for file

Additional file 2: Table S2 Ku70 is exclusively upregulated in IMR90-RSH cells but not IMR90 control cells. Click here for file

Additional file 3: Table S3 Genes related to DNA damage response in IMR90 RSH and IMR90 hTERT cells when compared to IMR90 control cells. Click here for file
