# Supplementary material for: The use of transformed IMR90 cell model to identify the potential extra-telomeric effects of hTERT in cell migration and DNA damage response
Source: BMC Biochem. 2014 Aug 7;15:17. doi: 10.1186/1471-2091-15-17 (PMC4126993; doi:10.1186/1471-2091-15-17)
Supplement: Additional file 1: Table S1 — Summary of recurrent molecular cytogenetic abnormalities detected in IMR90 RSH cells. [file 1471-2091-15-17-S1.docx]

**Table S1: Summary of recurrent molecular cytogenetic abnormalities detected in IMR90 RSH cells**

| **Cell No.** | **Abnormal karyotype result** |
| --- | --- |
| 1 | 46,XX, der(14)t(14;20)(p11;p11?), +der(18), der(19)t(19;20)(q13;q11), -20 |
| 2 | 45, XX, -4, del(5)(p14pter), der(16)ins(16;18)(q12;?), -16, der(18)t(4;18)(q21;q21), der(20)t(10;16;20) |
| 3 | 45, XX, -4, der(16)ins(16;18)(q12;?), der(18)t(4;18)(q21;q21), der(20)t(16;20)(q11;qter) |
| 4 | 45, XX, -4, der(16)ins(16;18)(q12;?), der(18)t(4;18)(q21;q21), der(20)t(10;16;20) |
| 5 | 45, XX, -4, der(16)ins(16;18)(q12;?), der(18)t(4;18)(q21;q21), der(20)t(10;16;20) |
| 6 | 45, XX, -4, +der(9), der(16)ins(16;18)(q12;?), der(18)t(4;18)(q21;q21), der(20)t(10;16;20),-22 |
| 7 | 45, XX, -4, der(13)t(13;16)(p11;p11), der(16)ins(16;18)(q12;?), der(18)t(4;18)(q21;q21), der(20)t(10;16;20) |
| 8 | 45, XX, -4, der(16)ins(16;18)(q12;?), der(18)t(4;18)(q21;q21), der(20)t(16;20)(q11;qter) |
| 9 | 45, XX, -4, der(16)ins(16;18)(q12;?), der(18)t(4;18)(q21;q21), der(20)t(16;20)(q11;qter) |
| 10 | 47, XX, +9, del(16)(p12pter), der(18)t(13;18) |
| 11 | 45, XX, -4, t(12;12)(q13;qter), der(16)ins(16;18)(q12;?), der(18)t(4;18)(q21;q21), der(20)t(16;20) (q11;qter) |
| 12 | 43, XX, der(2)dic(2;8)(q21;qter), -4, del(4)(q?), -8, -12, der(16)ins(16;18)(q12;?), der(18)t(4;18)(q21;q21), der(20)t(16;20) (q11;qter) |
| 13 | 45, XX, -4, der(13)t(13;16)(p11;p11), der(16) ins(16;18)(q12;?), der(18)t(4;18)(q21;q21), der(20)t(16;20)(q11;qter) |
| 14 | 45, XX, -4, der(16)ins(16;18)(q12;?), der(18)t(4;18)(q21;q21), der(20)t(16;20)(q11;qter) |
