# Supplementary material for: The use of transformed IMR90 cell model to identify the potential extra-telomeric effects of hTERT in cell migration and DNA damage response
Source: BMC Biochem. 2014 Aug 7;15:17. doi: 10.1186/1471-2091-15-17 (PMC4126993; doi:10.1186/1471-2091-15-17)
Supplement: Additional file 2: Table S2 — Ku70 is exclusively upregulated in IMR90-RSH cells but not IMR90 control cells. [file 1471-2091-15-17-S2.docx]

**Table S2: Ku70 is exclusively upregulated in IMR90-RSH cells but not IMR90 control cells**

Table S2a: Proteins detected by ESI-QUAD-TOF in the band of IMR90 control cells

| **prot_hit_num** | **prot_acc** | **prot_desc** | **prot_score** | **prot_mass** | **prot_pi** | **pep_seq** |
| --- | --- | --- | --- | --- | --- | --- |
| 1 | ANXA6 | Annexin A6 | 644 | 76168 | 5.42 | VFQEFIK |
|  |  |  |  |  |  | ALIEILATR |
|  |  |  |  |  |  | LVFDEYLK |
|  |  |  |  |  |  | SEIDLLNIR |
|  |  |  |  |  |  | CLIEILASR |
|  |  |  |  |  |  | SELDMLDIR |
|  |  |  |  |  |  | EAILDIITSR |
|  |  |  |  |  |  | DAFVAIVQSVK |
|  |  |  |  |  |  | SEIDLLNIRR |
|  |  |  |  |  |  | SLEDALSSDTSGHFR |
|  |  |  |  |  |  | LILGLMMPPAHYDAK |
|  |  |  |  |  |  | SLHQAIEGDTSGDFLK |
|  |  |  |  |  |  | GFGSDKEAILDIITSR |
|  |  |  |  |  |  | DLEADIIGDTSGHFQK |
|  |  |  |  |  |  | GLGTDEDTIIDIITHR |
|  |  |  |  |  |  | DLMTDLKSEISGDLAR |
|  |  |  |  |  |  | WGTDEAQFIYILGNR |
|  |  |  |  |  |  | GSIHDFPGFDPNQDAEALYTAMK |
| 2 | HSP7C | Heat shock cognate  71 kDa protein | 309 | 71082 | 5.37 | LLQDFFNGK |
|  |  |  |  |  |  | DAGTIAGLNVLR |
|  |  |  |  |  |  | FEELNADLFR |
|  |  |  |  |  |  | ARFEELNADLFR |
|  |  |  |  |  |  | SFYPEEVSSMVLTK |
|  |  |  |  |  |  | IINEPTAAAIAYGLDK |
|  |  |  |  |  |  | IINEPTAAAIAYGLDKK |
|  |  |  |  |  |  | TVTNAVVTVPAYFNDSQR |
| 3 | HSP71 | Heat shock 70 kDa protein 1 | 121 | 70280 | 5.48 | DAGVIAGLNVLR |
|  |  |  |  |  |  | IINEPTAAAIAYGLDR |
| 4 | ALBU | Serum albumin precursor | 115 | 71317 | 5.92 | LVTDLTK |
|  |  |  |  |  |  | YLYEIAR |
|  |  |  |  |  |  | KVPQVSTPTLVEVSR |

Table S2b. Proteins detected by ESI-QUAD-TOF in the band of IMR90 RSH cells

| **prot_hit_num** | **prot_acc** | **prot_desc** | **prot_score** | **prot_mass** | **prot_pi** | **pep_seq** |
| --- | --- | --- | --- | --- | --- | --- |
| 1 | HSP7C | Heat shock cognate 71 kDa protein | 381 | 71082 | 5.37 | LLQDFFNGK |
|  |  |  |  |  |  | DAGTIAGLNVLR |
|  |  |  |  |  |  | NSLESYAFNMK |
|  |  |  |  |  |  | ARFEELNADLFR |
|  |  |  |  |  |  | TTPSYVAFTDTER |
|  |  |  |  |  |  | SFYPEEVSSMVLTK |
|  |  |  |  |  |  | IINEPTAAAIAYGLDK |
|  |  |  |  |  |  | NQVAMNPTNTVFDAK |
|  |  |  |  |  |  | STAGDTHLGGEDFDNR |
|  |  |  |  |  |  | IINEPTAAAIAYGLDKK |
|  |  |  |  |  |  | TVTNAVVTVPAYFNDSQR |
| 2 | ALBU | Serum albumin precursor | 294 | 71317 | 5.92 | AACLLPK |
|  |  |  |  |  |  | LVTDLTK |
|  |  |  |  |  |  | YLYEIAR |
|  |  |  |  |  |  | YICENQDSISSK |
|  |  |  |  |  |  | VPQVSTPTLVEVSR |
|  |  |  |  |  |  | KVPQVSTPTLVEVSR |
|  |  |  |  |  |  | RPCFSALEVDETYVPK |
| 3 | ANXA6 | Annexin A6 | 278 | 76168 | 5.42 | ALIEILATR |
|  |  |  |  |  |  | LVFDEYLK |
|  |  |  |  |  |  | SEIDLLNIR |
|  |  |  |  |  |  | SELDMLDIR |
|  |  |  |  |  |  | DAFVAIVQSVK |
|  |  |  |  |  |  | SLEDALSSDTSGHFR |
|  |  |  |  |  |  | LILGLMMPPAHYDAK |
|  |  |  |  |  |  | SLHQAIEGDTSGDFLK |
| 4 | HSP71 | Heat shock 70 kDa protein 1 | 248 | 70280 | 5.48 | DAGVIAGLNVLR |
|  |  |  |  |  |  | NALESYAFNMK |
|  |  |  |  |  |  | AQIHDLVLVGGSTR |
|  |  |  |  |  |  | TTPSYVAFTDTER |
|  |  |  |  |  |  | AFYPEEISSMVLTK |
|  |  |  |  |  |  | IINEPTAAAIAYGLDR |
| 5 | KU70 | ATP-dependent DNA helicase 2 subunit 1 | 104 | 70084 | 6.23 | ILELDQFK |
|  |  |  |  |  |  | KQELLEALTK |
|  |  |  |  |  |  | DSLIFLVDASK |
|  |  |  |  |  |  | SDSFENPVLQQHFR |
|  |  |  |  |  |  | TFNTSTGGLLLPSDTKR |
