# Supplementary material for: The use of transformed IMR90 cell model to identify the potential extra-telomeric effects of hTERT in cell migration and DNA damage response
Source: BMC Biochem. 2014 Aug 7;15:17. doi: 10.1186/1471-2091-15-17 (PMC4126993; doi:10.1186/1471-2091-15-17)
Supplement: Additional file 3: Table S3 — Genes related to DNA damage response in IMR90 RSH and IMR90 hTERT cells when compared to IMR90 control cells. [file 1471-2091-15-17-S3.docx]

**Table S3: Genes related to DNA damage response in IMR90 RSH and IMR90 hTERT cells when compared to IMR90 control cells**

| **GO Function** | **Gene** | **Chr #** | **Gene Annotation** | **Fold-Change**  **IMR90 hTERT vs control** | **Fold-Change**  **IMR90 RSH**  **vs control** |
| --- | --- | --- | --- | --- | --- |
| DNA Damage Repair Associated | MDC1 | 6 | Mediator of DNA damage checkpoint 1 | 4.090 | 2.481 |
|  | LIG1 | 19 | Ligase I, DNA, ATP-dependent | 3.278 | 2.179 |
|  | TOPBP1 | 3 | Topoisomerase (DNA) II binding protein 1 | 2.923 | 2.048 |
|  | PRKDC | 8 | Protein kinase, DNA-activated, catalytic polypeptide | 3.337 | 2.024 |
|  | DDIT4 | 10 | DNA-damage-inducible transcript 4 | 2.758 | 8.010 |
|  | TOP2A | 17 | Topoisomerase (DNA) II alpha 170kDa | 11.07 | 6.989 |
|  | DEK | 6 | DEK oncogene (DNA binding) | 4.705 | 4.077 |
|  | DNMT1 | 19 | DNA (cytosine-5-)-methyltransferase 1 | 6.223 | 3.812 |
|  | POLD1 | 19 | Polymerase (DNA directed), delta 1, catalytic subunit | 2.607 | 2.780 |
|  | COBRA1 | 9 | Cofactor of BRCA1 | 3.897 | 1.990 |
|  | DNMT1 | 19 | DNA (cytosine-5-)-methyltransferase 1 | 6.223 | 3.812 |
|  | RAD21 | 8 | RAD21 homolog (S. pombe) | 2.039 | 1.798 |
|  | RAD51 AP1 | 12 | RAD51 associated protein 1 | 3.522 | 2.396 |
|  | RAD23A | 19 | RAD23 homolog A (S. cerevisiae) | 3.131 | 2.131 |
